# Effects of Landscape Type Change on Spatial and Temporal Evolution of Ecological Assets in a Karst Plateau-Mountain Area

**DOI:** 10.3390/ijerph19084477

**Published:** 2022-04-08

**Authors:** Cheng He, Kangning Xiong, Yongkuan Chi, Shuzhen Song, Jinzhong Fang, Shuyu He

**Affiliations:** 1 School of Karst Science, Guizhou Normal University, Guiyang 550001, China; hecheng25@163.com (C.H.); hebeichiyongkuan@163.com (Y.C.); gysongsz@sina.com (S.S.); fjinz1314@163.com (J.F.); heshuyu13550048382@163.com (S.H.); 2 State Engineering Technology Institute for Karst Desertification Control, Guiyang 550001, China

**Keywords:** karst plateau-mountains, landscape types change, ecological assets, spatial and temporal evolution

## Abstract

The rocky desertification control project in karst areas exacerbates the transfer of landscape types, changes the ecosystem structure and function, and has a significant impact on ecological assets. How to analyze the relationship between landscape type shifts and the spatial and temporal evolution of ecological assets is one of the key questions that need to be addressed to achieve the goal of overall improvement in ecosystem quality and sustainable regional economic development. This study takes Qixingguan District, Bijie City, Guizhou Province—a typical karst plateau mountainous area—as the research object, and analyzes the spatial and temporal evolution characteristics of landscape type shifts and ecological assets triggered by rock desertification management from 1995–2018, based on the equivalence factor method, combined with the contribution rate, spatial autocorrelation, and sensitivity research methods. The results showed that arable land, grassland, and woodland were the main landscape types in the study area. The value of ecological assets showed a trend of increasing and then decreasing, with an overall increase of 87.70 × 10^6^ yuan. The distribution pattern of ecological asset value from southwest to northeast is “high–low–high”. There is a significant positive correlation in the spatial distribution of the overall ecological assets, with similar aggregation between neighboring units. The expansion of forest land was the main factor for the rapid increase of assets from 1995 to 2010, with a contribution of 98.12%; the conversion of arable land and grassland to construction land was the main factor for the decrease of assets from 2010 to 2018, with a percentage of 81.06%, where the value of each type of service was mainly composed of five items, such as soil formation and conservation, biodiversity conservation and gas regulation, water conservation, and climate regulation. This study shows that spatial and temporal evolution assessment of ecological assets is an important manifestation of the effectiveness of rocky desertification control, which can provide decision support to resource managers and users for regional ecological environment construction.

## 1. Introduction

Karst is an ecologically fragile area of the most typical, concentrated, and comprehensive geomorphological type development in the world, it is a distribution pattern centered on the Guizhou plateau and spreads outward, with a rocky exposed area of about 5.5 × 10^5^ km^2^ [1]. Although providing rich economic assets and unique habitats, these are affected by both natural factors and human economic activities, leading to soil erosion, biodiversity loss and ecosystem function decline [2], all of which seriously threaten regional ecological environmental security and constrain sustainable socio-economic development [3]. It takes thousands of years to form a stable karst system, and damage to natural resources and ecosystem services from climate change, human activities, and environmental pollution is difficult to restore [4]. With the increased awareness of the complexity and vulnerability of karst areas, a series of rock desertification management projects have been carried out to reduce intensive human use of land, and the expansion of rock desertification area, vegetation destruction and soil degradation have been initially alleviated [5]. Since ecological assets are the comprehensive embodiment of natural resource attributes and ecosystem service attributes of ecosystems, which provide the corresponding basis for karst ecological function area planning and ecosystem protection [6], it is urgent to carry out regional ecological assets research.

Ecological assets are ecosystems that provide ecological products and service benefits to humans in a certain spatial and temporal context with technical and economic conditions, including two categories of direct ecological product values (supply service values) and indirect ecosystem service values (regulation, support, and cultural service values) [7,8,9,10], and the value connotations of ecological assets and ecosystem services tend to be homogenized as domestic and foreign. This leads to the analysis of the impact of land use and multilevel climate transformation on the value of global ecosystem services under different scenarios [11,12]. Subsequently, Xie et al. proposed the equivalence factor method considering the actual situation in China to solve the problem of large errors in accounting for medium-scale ecosystem services, and developed a table of value coefficients per unit area for terrestrial ecosystem services in China which was divided into nine categories: gas regulation, climate regulation, water conservation, soil formation and protection, waste disposal, biodiversity conservation, food production, raw material, and entertainment culture [13]. With the rise of remote sensing technology, the study of regional ecological assets has moved from static to dynamic assessment, from overall value estimation to the analysis of ecosystem type transformation, quality status and spatial and temporal evolution of ecological assets [14,15]. Due to the strong spatial heterogeneity in karst regions, numerous factors influence the fluctuating changes in ecological asset values, with climate and landscape type changes as the main factors driving changes in spatial and temporal patterns of ecological assets in these karst regions, where landscape type changes are the main control factors that anthropogenically cause spatial and temporal differences in regional ecological assets [16,17]. Affecting the main ecological processes are factors such as energy exchange, water cycle, and biochemical cycle of ecosystems, changing the value of individual service functions such as terrestrial water cycle, biodiversity, soil erosion and soil organic carbon, and total regional ecological services [18,19,20,21,22]; only by rational landscape planning can it promote coordinated regional economic-social-ecological development.

This paper applies the equivalence factor method, revises the Chinese ecosystem service value coefficient table, analyzes the land use data from 1995–2018 by remote sensing and GIS technology, explores the process of landscape type transfer in karst plateau mountains, elucidates the spatial and temporal evolution characteristics of regional ecological assets, and has important indicative significance for ecosystem protection and restoration in karst rocky desertification areas, with a view to providing a scientific basis for global karst ecological environment construction and regional economic development.

## 2. Materials and Methods

### 2.1. Study Area

**Figure 1:**
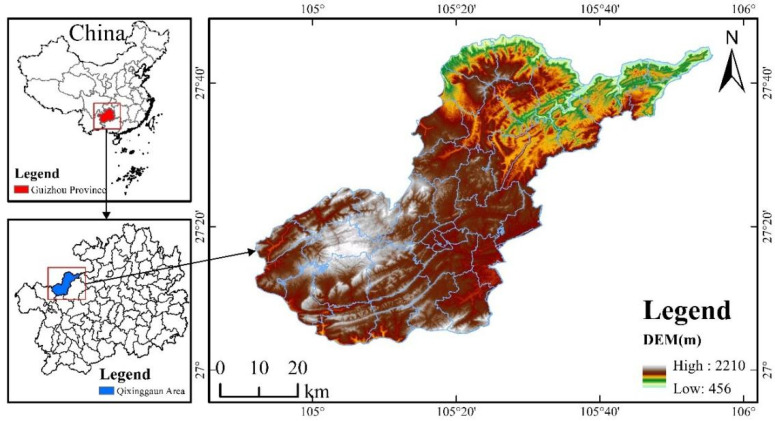
The location of study area.

The study area is located in Qixingguan District, Bijie City, Guizhou Province (104°52′–105°56′ E, 27°03′–27°46′ N), a central city at the junction of Sichuan, Yunnan and Qian in the northern part of the Yunnan–Guizhou Plateau, covering an area of about 3412 km^2^ (Figure 1). The limestone karst landform types, such as peaks, water caves, and dissolved depressions are widely distributed in the area. The terrain is high in the west and low in the east, with a step-like descent from southwest to northeast and an altitude difference of 1754 m. It belongs to the subtropical humid monsoon climate type, with no severe cold in winter and summer heat, the average annual temperature is about 13.6 °C, the average annual relative humidity is about 80.8%, the average annual precipitation is about 1126.9 mm, and the average annual sunshine hours are 1178 h. Soil types are dominated by limestone, loam, and yellow-brown loam [23]. The unique topographic features and hydrological processes cause weak structural stability of the ecosystem, forming a typical ecologically fragile karst area susceptible to disturbance and with poor resilience.

### 2.2. Data Sources

The data used in this paper include: (1) land use data: six periods of land use data (1995, 2000, 2005, 2010, 2015, and 2018) from the Resource and Environment Science and Data Center of the Chinese Academy of Sciences (https://www.resdc.cn/ (accessed on 10 September 2021)) with a resolution of 30 m and a decoding accuracy of more than 90% [24]. (2) Topographic data: DEM30 m digital elevation data of Guizhou Province from the website of “Geospatial Data Cloud” (https://www.gscloud.cn/ (accessed on 15 September 2021)). (3) Socio-economic data: from the China Statistical Yearbook (http://www.stats.gov.cn/tjsj/ndsj/ (accessed on 23 September 2021)), the statistical bulletin on national economic and social development of Qixingguan District People’s Government of Bijie City (http://www.bjqixingguan.gov.cn/ (accessed on 11 October 2021)), and the China Agricultural Information Network (http://www.agri.cn/ (accessed on 15 October 2021)) for annual production, sown area, and prices of major grains (corn, wheat, and rice).

### 2.3. Research Methodology

#### 2.3.1. Research Methodology Flowchart

**Figure 2:**
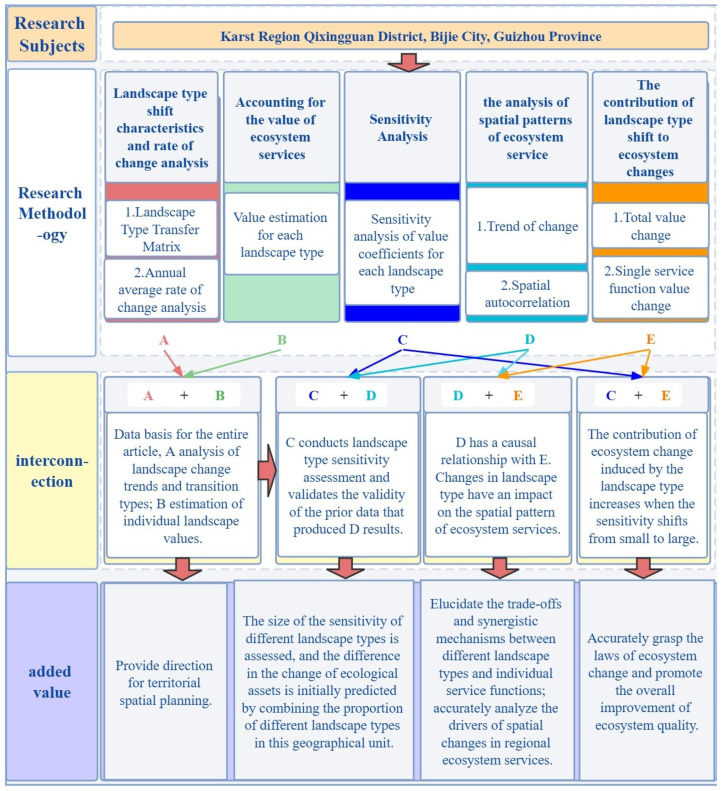
Research Methodology Flowchart. Note: A, B, C, D and E in the figure represent Landscape type shift characteristics and rate of change analysis, Accounting for the value of ecosystem services, Sensitivity Analysis, the analysis of spatial patterns of ecosystem service, The contribution of landscape type shift to ecosystem changes.

As shown in Figure 2, the methodological flow of this paper is to identify the research object, clarify the interconnection between different research methods, and elucidate their added value.

#### 2.3.2. Landscape Type Transition Characteristics and Rate of Change Analysis

The average annual rate of change equation measures the rate of change of a single landscape type and is important for measuring its spatial and temporal variability [25], as follows:

(1) $$K=\frac{U_{B}-U_{A}}{U_{A}}\times \frac{1}{T}\times 100\%\;$$

where K is the average annual rate of change, $U_{A}$ and $U_{B}$ are the area of a type of land before and after the study, respectively, and *T* is the time span of the study period.

The landscape type transfer matrix fully characterizes the dynamic evolutionary process of interconversion between various land types under a certain spatial and temporal condition [26], the reclassification was performed using AcrMap10.2 analysis software, resulting in six categories: arable land, woodland, grassland, waters, construction land, and unused land. The spatial analysis function in the software was then used to establish the landscape type transfer matrix. The values 1–6 were assigned to arable land, forest land, grassland, water bodies, construction land, and unused land, respectively [27], and the specific formulas are as follows:

(2) N=10A+B

where N is the new map unit of landscape type transfer, A is the map unit of the pre-study period, and B is the map unit of the end of the study period.

#### 2.3.3. Estimating the Value of Ecosystem Services

The standard equivalence factor was revised using the actual biomass of farmland in the study area [28], based on which a correction factor of 0.619 for the standard equivalence factor was derived as the ratio of the average grain yield of 3230.52 kg/hm^2^ in the study area to the national average grain yield of 5233.15 kg/hm^2^. To reduce the disturbance of spatial and temporal heterogeneity and socioeconomic fluctuations, the average food production and prices in the study area from 1995–2018 were used as the benchmark for analysis to accurately assess the impact of landscape type transfer on ecosystem service values (ESVs). Using rice wheat corn as the main grain price for calculation, a national standard equivalence factor for ESVs was derived as 2280.16 Yuan/hm^2^·a. Based on the table of Chinese terrestrial ESVs equivalence factors, a table of ESVs factors per unit area in the study area was converted by incorporating correction factors (Table 1), where the value coefficient of construction land is zero.

The ESVs are calculated as follows [13,29]:

(3) $$E_{a}=\frac{1}{7}W_{av}\;$$

(4) $$VC_{ij}=EC_{ij}\times E_{a}$$

(5) $$ESV=\overset{n}{\underset{i=1}{\sum }}\left(A_{i}\times \overset{k}{\underset{j=1}{\sum }}VC_{ij}\right)$$

where $E_{a}$ is the national standard equivalent ecosystem service value, $W_{av}$ is the average annual price of food per unit area in the study area (Yuan/hm^2^·a), $VC_{ij}$ is the value coefficient of ecosystem service function type j of type i landscape, $EC_{ij}$ is the value equivalence factor of ecosystem service function j of type i landscape after correction, ESVs are ecosystem service values, and $A_{i}$ is the area of type i landscape.

#### 2.3.4. Sensitivity Analysis

To avoid fluctuations in the accounting results of ecosystem service value coefficients from affecting the accuracy judgment, a sensitivity index was applied to determine the dependence of ESVs on ecosystem service value coefficients over time [30]. The expressions are as follows:

(6) $$CS=\left|\frac{ESV_{B}-ESV_{A}/ESV_{A}}{VC_{Bi}-VC_{Ai}/VC_{Ai}}\right|$$

where CS is the sensitivity coefficient, $ESV_{A}$ and $ESV_{B}$ are the total value of ecosystem services before and after adjustment, and $VC_{Ai}$ and $VC_{Bi}$ represent the ecosystem service value coefficients per unit area of ecosystem type i before and after adjustment. The value coefficient of ecosystem services VC was calculated by increasing or decreasing the value coefficient of each landscape type by 50%, respectively. If CS> 1, it means that ESV is elastic to VC, the value coefficient has low credibility; conversely, it is inelastic, and the result is credible.

#### 2.3.5. Spatial Patterns of Ecosystem Service Values

(1) Trends in spatial pattern changes

The spatial layout of the value of ecosystem services is described in the paper using townships as the study unit, and the amount of value change is expressed using CV, calculated as follows:

(7) $$CV_{i}=ESV_{end}-ESV_{start}$$

where $CV_{i}$ denotes the change in ecosystem service value of different study units, $ESV_{end}$ is the ESV at the end of the study period; $ESV_{start}$ is the ESV at the beginning of the study period.

(2) Spatial autocorrelation analysis

Spatial autocorrelation analysis is a measure of whether the distribution of spatial variables is clustered and contains both global spatial autocorrelation and local spatial autocorrelation [31], which was performed using GeoDa 1.1.2 software.

Global spatial autocorrelation reveals the spatial correlation of unit attribute values with neighboring units. The global Moran’s I is the widely used global autocorrelation statistical measure and is calculated as follows [32]:

(8) $$I=\frac{\sum_{i}^{n}\sum_{j}^{n}w_{ij}\left(x_{i}-\bar{x}\right)\left(x_{j}-\bar{x}\right)}{S^{2}\left(\sum_{i}\sum_{j}w_{ij}\right)}$$

Local Indicators of Spatial Association (LISA) is often measured using the local Moran’s I statistic, which measures the degree of similarity or difference between the observed unit attribute values and the surrounding unit attribute values, and is plotted on the basis of the z-test (*p* < 0.05) LISA distribution plot, calculated as follows:

(9) $$I_{i}=\frac{\left(x_{i}-\bar{x}\right)\sum_{j=1}^{n}W_{ij}\left(x_{i}-\bar{x}\right)}{S^{2}}$$

where *n* is the number of spatial cells, $x_{i}$ and $x_{j}$ denote the observations of cell i and cell j, respectively, $\left(x_{i}-\bar{x}\right)$ is the deviation of the observation on the ith spatial cell from the mean, $W_{ij}$ is the spatial weight matrix based on the spatial k neighborhood, and the variance $S^{2}=\frac{1}{n}\sum_{i=1}^{n}{\left(x_{i}-\bar{x}\right)}^{2}$.

#### 2.3.6. The Contribution of Landscape Type Shift to Ecosystem Changes

Analysis of the extent to which the transfer of different landscape types affects the value of ecosystem services was performed using an analysis of the value generated by the transformation between different landscapes versus the proportion of the total value generated by the transformation in that landscape, with the following equation [33]:

(10) $$EL_{i-e}=\frac{\Delta VC_{i-e}\times A_{i-e}}{\sum_{i=1}^{n}\left(\Delta VC_{i-e}\times A_{i-e}\right)}\times 100\%$$

where $EL_{i-e}$ is the contribution of landscape change from land class i to e to ecosystem service value, $\Delta VC_{i-e}$ is the amount of change in ecosystem service value coefficient from land class i to e, and $A_{iT}$ is the area of landscape transferred from class i to class e.

## 3. Results

### 3.1. Landscape Type Shift Characteristics and Rate of Change Analysis

**Figure 3:**
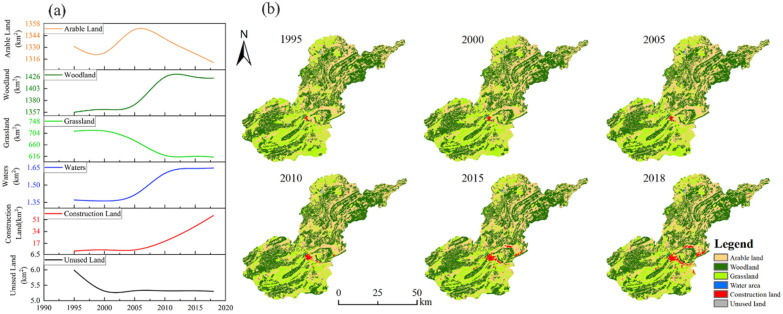
Land use change map (**a**) and stacked diagram (**b**) for the study area from 1995–2018.

From 1995–2018, arable land, woodland, and grassland were the dominant landscape types in the study area, spatially, with woodland, waters, and construction land areas showing an increasing trend (Figure 3).

**Figure 4:**
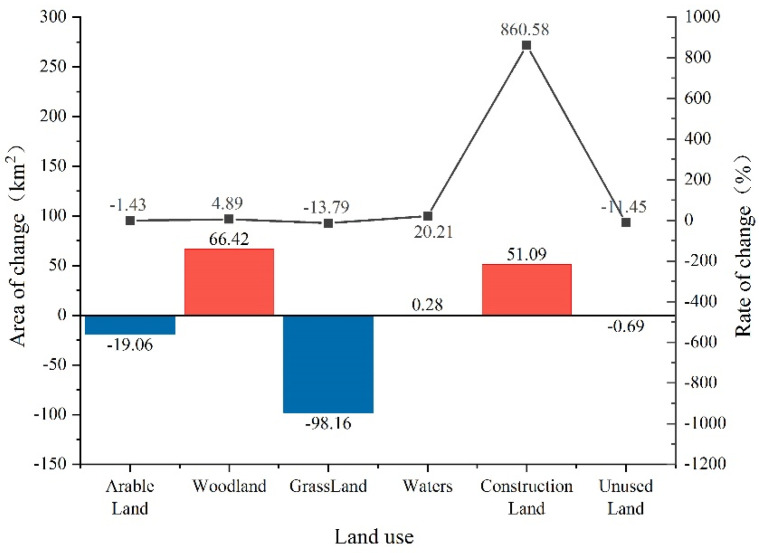
Landscape type area change and rate of change during 1995−2018.

During the study period, the area of forest land, construction land, and water land types increased significantly (Figure 4), and the change rate was construction land > water surface > forest land; the area of grassland, cropland, and unused land decreased significantly, and the change rate was grassland > unused land > cropland.

In Table 2, the average rate of change of grassland from 1995 to 2000 is 0.03 > 0, and the average rate of change of cropland from 2000 to 2005 is 0.42 > 0. The rest of the periods are negative, indicating that the area of cropland and grassland has an increasing trend only in one period; the average annual rate of change of cropland, grassland, and unused land over 23 years is negative, indicating that the area of these three landscapes as a whole has a decreasing trend, with grassland. The decrease is the most significant, with a change rate of −0.6%. The annual average rate of change of woodland in 2015–2018 and construction land in 2000–2005 were both −0.05% < 0, and the rest of the periods were positive, indicating that woodland and construction land only had a decreasing trend in one period; the annual average rate of change of woodland, water area, and construction land during the study period was positive, indicating that the overall trend of these three landscape areas was increasing, with construction land increasing the most. The rate of change was 37.42%. The area of different landscape types in the study area shows fluctuating rise and fall rather than linear change.

From 1995–2018, 25 landscape types were transferred in the study area (Table 3), with a total change area of 401.60 km^2^ and an area of 11.77%. Cultivated land has the largest area transferred in the process of landscape type transfer, accounting for 39% of the total change area, and the main types of transfer are woodland, grassland and construction land, with a significant increase in the area of woodland, accounting for 45.28% of the total area transferred from cultivated land; grassland follows in the transfer process, mainly transferred to woodland, cultivated land and construction land, with an increase in the area of woodland accounting for 53.84% of the total area transferred from grassland, with cultivated land. The interconversion activities between cropland and grassland were intense and the total area transferred was very close. Forest land is the main type of conversion from other land types, accounting for 38.51% of the total changed area, and the conversion of grassland to forest land is the most, accounting for 54.56% of the total converted area of forest land; the converted area of cropland is the second, accounting for 34.23% of the total changed area, of which the converted area of forest land to cropland is the most, with a conversion rate of 54.72%.

**Figure 5:**
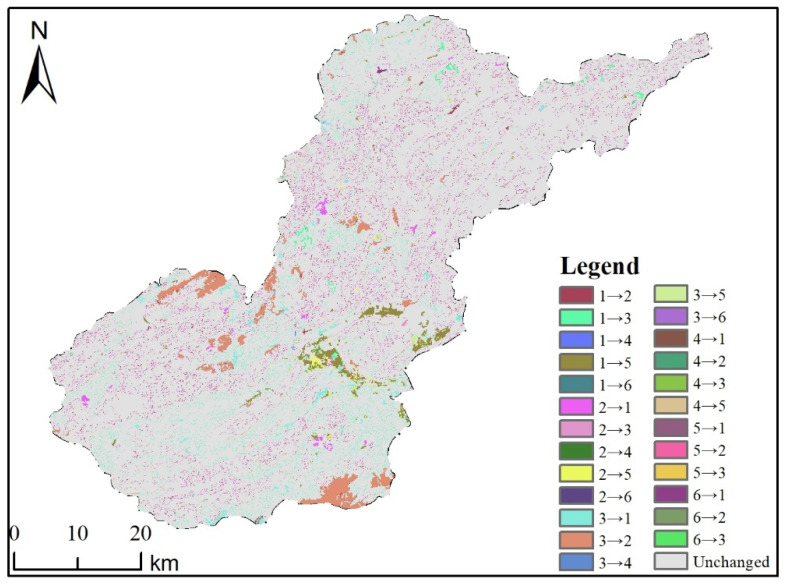
Spatial distribution map of mutual transfers by land use types in the study area from 1995–2018. Note: 1, 2, 3, 4, 5, and 6 in the figure represent arable land, woodland, grassland, waters, construction land, and unused land, respectively; 1→2 represents the conversion of arable land to woodland during the study period; Unchanged represents no land change has occurred.

As shown in (Figure 5), the middle part of the study area is the range where the most dramatic landscape type transfer occurs, and the mutual conversion of cropland to forest land and grassland mainly occurs, mainly in the way of grassland to forest land, forest land to cropland and cropland to construction land.

### 3.2. Accounting for the Value of Ecosystem Services

**Figure 6:**
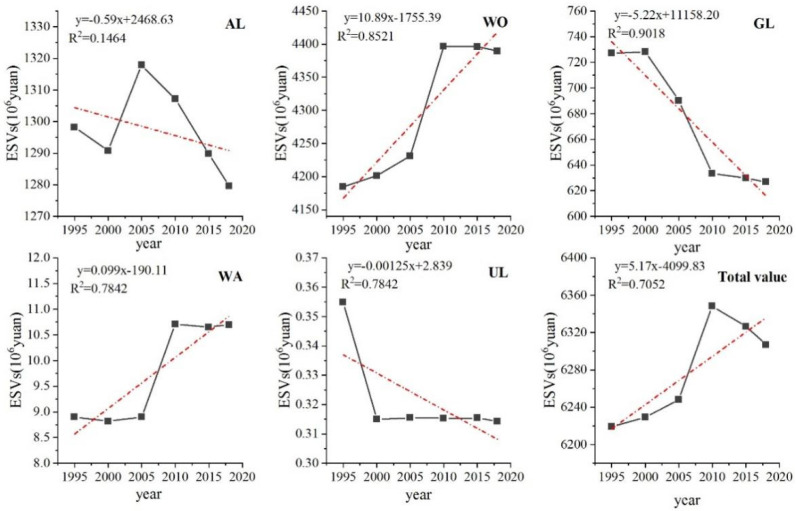
ESVs and total value of various land use types in the study area during 1995−2018. Note: AL, WO, GL, WA, and UL represent arable land, woodland, grassland, water area and unused land in the figure.

Under the influence of the overall landscape type shift, as shown in (Figure 6), the change of ecosystem service value varies among different landscape types. The value of cropland fluctuates most sharply, showing a “decrease–increase–decrease” trend, with ESVs decreasing by 7.36 × 10^6^ yuan before 2000, increasing by 27.09 × 10^6^ yuan in the next 5 years, and decreasing by 38.32 × 10^6^ yuan after 2005, for a cumulative decrease in total ESVs of 18.59 × 10^6^ yuan. The change trend of increasing ESVs was similar for woodland and watershed, but from 2010 to 2018 woodland ESVs decreased by 7.14 × 10^6^ yuan and watershed remained largely unchanged, with woodland and watershed ESVs increasing by 211.36 × 10^6^ yuan and 1.8 × 10^6^ yuan, respectively, during the study period. Grassland ESVs showed a continuous decreasing trend with a cumulative decrease in ESVs of 100.3 × 10^6^ yuan. The decrease in ESVs of unused land maintained a stable state until 2000, with a decrease of 0.04 × 10^6^ yuan.

The trend of the total ecosystem service value in the study area was to increase first and then decrease, and the increase of the service value of forest land and cropland was much higher than the decrease of grassland up to 2010, showing a significant increase trend, and the total ESVs increased by 129.02 × 10^6^ Yuan, an increase of 2.7%; after that, cropland, forest land and grassland all showed a decrease, much higher than the increase of water bodies, showing a gentle decreasing trend, with a total decrease of 41.32 × 10^6^ yuan and a decrease rate of 0.65%. The total value of ecosystem services increased by 87.70 × 10^6^ yuan during the study period, with a growth rate of 1.41%. The period between 2005 and 2010 was the period of more drastic changes in the value of forest land, grassland, watershed, and cropland, and also the period of the largest increase in total ESVs. In conclusion, the increase in the area of forest land was the main reason for the increase in value until 2010; after that, the decrease in the area of grassland and cropland was the main reason for the decrease in value.

### 3.3. Sensitivity Analysis

**Figure 7:**
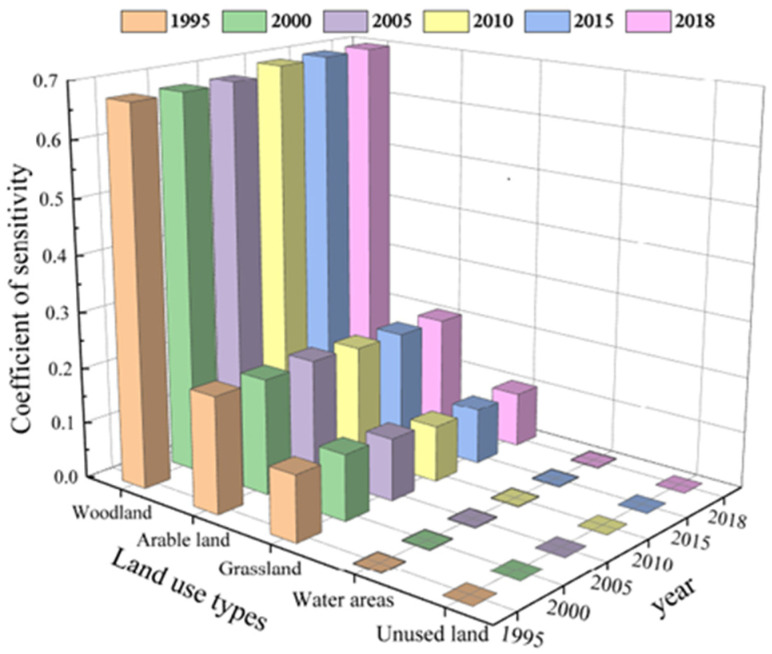
Coefficient of sensitivity of ecosystem services value.

As shown in (Figure 7), for different ecosystem service value coefficients increased or decreased by 50%, the overall sensitivity index was still <1 and the degree of change was relatively small. The maximum value of sensitivity among different landscape types is forest land, i.e., the corresponding value ± (0.6728–0.69598%) when the value coefficient of forest land is adjusted to ±1%, which reveals that the highly resilient forest land type in the study area shifts rapidly, with unstable transfers in and out and drastic changes in system structure. The minimum value is unused land with a value range of 0.00005–0.00006; the coefficient is adjusted by ±1% for ±(0.00005–0.00006%) value change. The overall sensitivities are forest land > cropland > grassland > watershed > unused land from the largest to the smallest, and the results of the study indicate that the ecosystem service value coefficient is inelastic, and its results are true and valid.

### 3.4. Spatial and Temporal Distribution of Ecological Asset Values

#### 3.4.1. Trend of Change

**Figure 8:**
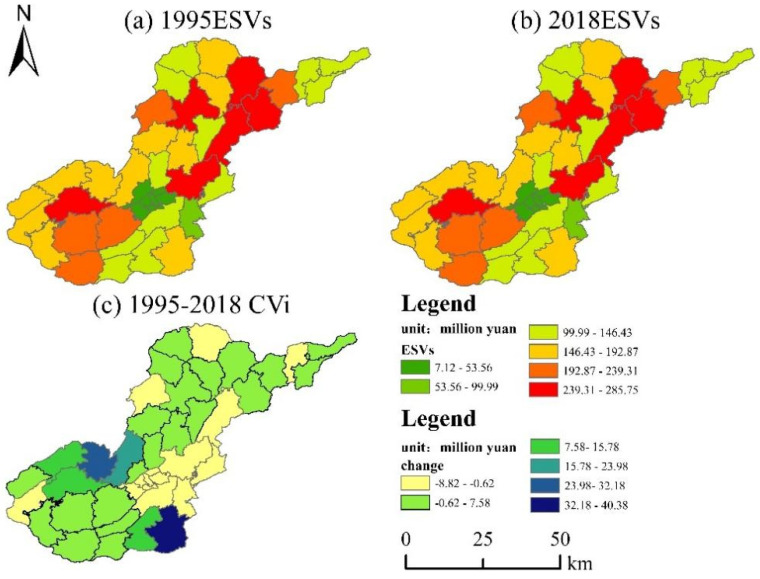
Spatial distributions of ESVs in the study area in 1995 (**a**) and 2018 (**b**) and the spatial distribution of CVi (**c**) in the study area during 1995–2018.

The spatial and temporal trends of ESVs are characterized by using township geographical units, and the service values per unit area are graded at equal intervals by GIS spatial statistical techniques, divided into six stages, and graded by specific color fields to present the differential changes of ecosystem service values of different units. From 1995 to 2018, the ESVs in the northeastern part of the study area were higher (Figure 8a,b), and the overall ESVs showed a “U” spatial distribution, with a “high–low–high” spatial distribution pattern from the southwest to the northeast. The overall ESVs show a “U” spatial distribution, with a “high–low–high” spatial distribution pattern from southwest to northeast. Although the overall ecosystem service value changes in the study area showed an increasing trend, a decreasing trend in ESVs was observed in some areas based on the difference in changes in (Figure 8c), and these townships were concentrated in urban center areas, such as Xiaoba, Yachi, and Liucangqiao, which caused a decrease in value due to the urbanization process occupying higher value coefficients in the upland category. The increase in ESVs is mainly distributed in Chahe, Shuiqing and Heguantun, which are spatially distributed far from the central city, and the landscape change is generally shifted to a higher value coefficient category.

#### 3.4.2. Spatial Autocorrelation Analysis of Ecosystem Service Values

(1) Global spatial autocorrelation

Spatial autocorrelation analysis of ecosystem service values from 1995–2018 revealed that the global Moran’s I values for the study area were all greater than 0.48 (*p* < 0.05) (Table 4), ESVs in response to landscape change showed significant spatial clustering across the study units as a whole, with a highly significant positive correlation between the units The relationship between the ESVs in response to land use change and the ESVs in the study units showed significant spatial clustering and a highly significant positive correlation among the units.

(2) Local spatial autocorrelation

**Figure 9:**
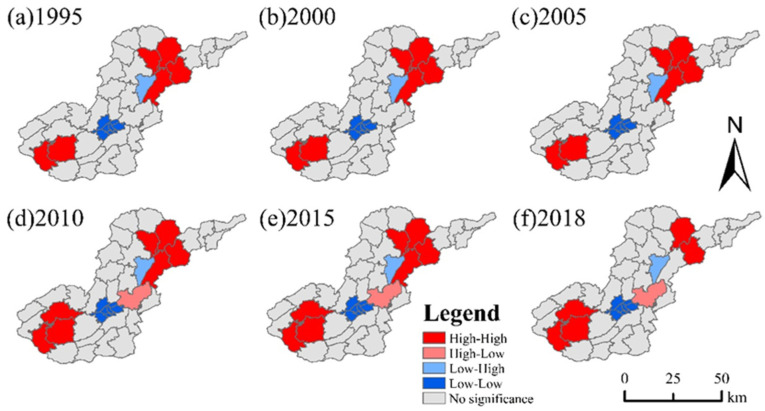
LISA distribution map of the ESVs in the study area in 1995 (**a**), 2000 (**b**), 2005 (**c**), 2010 (**d**), 2015 (**e**), and 2018 (**f**).

The spatial distribution of EVSs has a large and significant correlation, and local spatial autocorrelation analysis was carried out to clarify the types and specific locations of regional aggregation characteristics. From (Figure 9) local spatial autocorrelation Lisa plots, it is clear that the local aggregation of ESVs did not change from 1995 to 2005, and the local (H-H) agglomerative geographic units of ESVs are located in the southwest and northeast ends of the study area, and these areas will influence each other positively; the local (L-L) agglomerative geographic units of ESVs are in the central part of the study area, such as Liucangqiao, Shixi, Shidong, the Guanyinqiao and Sanbanqiao urban center areas will have a negative influence.

From 2010 to 2015, local (H-L) aggregation of ESVs occurred in the town of Cengtai and (L-H) in the town of Haizijie, and the trends of ESVs between the two geographical units were independent of each other, indicating that the autocorrelation of the local units was not significant. A shift in the local (H-H) aggregation of ESVs between Xiaojichang town and Liangyan town to non-significant occurred in 2018, indicating that this type of unit does not have spatial autocorrelation.

### 3.5. Value Contribution of Each Type of Service and Individual Service Function

**Figure 10:**
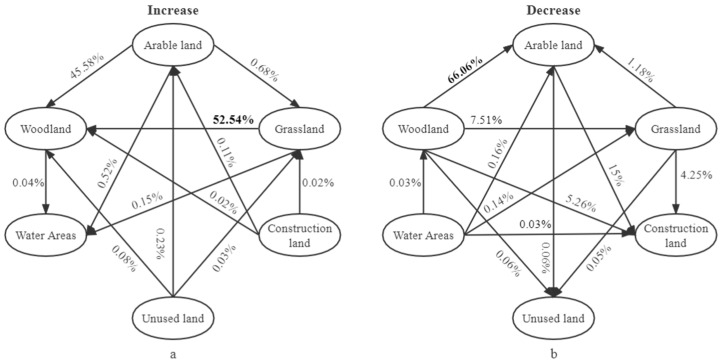
Contribution rate of different land use transfer types to the increase (**a**); and decrease (**b**) of ecosystem service value.

As shown in (Figure 10a), the 12 landscape shifts that occurred from 1995–2018 resulted in an increase in total ESVs of 328.11 × 10^6^ yuan, with the largest contribution from grassland to woodland, followed by cropland to woodland. Meanwhile, the 13 landscape shifts that occurred resulted in a decrease in total ESVs of 240.39 × 10^6^ yuan (Figure 10b), with the conversion of forest land to cropland being the most efficient in inhibiting value growth, followed by the conversion of cropland to building land. The results showed that the contribution of forest land expansion to the growth of total ESVs was 98.12%. Conversion of forest land to cropland was the main reason for the decrease of total ESVs, and conversion of cropland to construction land was the secondary reason for the decrease of total ESVs. The average contribution of each landscape type was in the following order: forestland > cropland > grassland > watershed > unused land.

From 1995–2018, the value of food production and waste treatment in single ecosystem service function (ESVf) showed a decreasing trend (Table 5); the value of the remaining functions showed an increasing trend, with the largest increase in the value of recreation and culture, followed by the value of raw materials. The trend of the ranking of its value composition did not change over 23 years, indicating that soil formation and conservation, biodiversity conservation, gas regulation, water conservation and climate regulation are still the main contributors to the value of ecological assets in karst stone desertification areas.

## 4. Discussion

The main landscape types in the study area are arable land, forest land, and grassland, which show the interconversion of arable land, forest land, and grassland, and the main transfer processes such as the conversion of arable land to construction land, which differ greatly from the results of global and national studies on the conversion of arable land to forest land, grassland, and construction land [34,35]. Limited by the inability of arable land resources to meet the food demand of a high-density population, irrational economic activities such as slash-and-burn farming and ore mining reduce forest and grass areas and destroy the ecological environment. In order to get rid of the poverty plight, the Chinese government launched the “Food Green Project” and started to return the cultivated land to forest and grass on a large scale, with corresponding ecological compensation [36]. The subsequent phase I of the rock desertification control project achieved results such as curbing the expansion of rock desertification, increasing vegetation cover, reducing pressure on natural ecosystems and improving ecosystem services [37,38], and increasing the area of forest and grass. As a result, ecological assets in karst areas showed an increasing trend from 1995 to 2010, and the transformation of arable land and grassland into forest land and water areas with higher value coefficients drove the value of ecological assets to increase. However, rapid urbanization, weak awareness of ecological safety among local residents, and lack of ecological compensation mechanisms caused continuous and dramatic alternating shifts in the 3 types of landscapes [39]. The trend of decrease from 2010 to 2018 is contradictory to the result that the rate of increase in ESVs reached the maximum [25,40], because the urbanization process prompted the conversion of sloping arable land and grassland together into construction land with a value coefficient of 0. Meanwhile, overgrazing behavior exceeded the ecological carrying capacity threshold and caused the degradation of grassland, while the ecological asset value of forest land changed slightly, and the area of arable land and grassland showed a trend of continuous decrease, resulting in the slow decrease of the overall ecological asset value in the study area [41]. Therefore, it is important to clarify the process of landscape type transfer of karst rock desertification management and to explore the spatial and temporal evolution characteristics of ecological assets to solve the problem of balanced development of economic, social, and ecological effects [42].

Ecological assets in the study area show a spatial distribution pattern of “high–low–high” from the northeast to the center of the city due to the expansion of urban land and rapid economic development, coupled with ecological migration driven by policy reasons [43], which together trigger population aggregation and dispersion resulting in a decline in ecological assets. The findings indicating a negative correlation between population density and the high value of ecological assets are consistent [44].

During the study period, the Moran’s I table of ESVs across the region changed, indicating that the regional ESVs suffered from external disturbance factors and the clustering stability was weak, and a large amount of resources were invested in implementing precise poverty alleviation policies to eliminate absolute poverty [45], which indirectly drove the transfer of ecological assets across the region between different township units, resulting in an unstable degree of global spatial correlation stability. This directly affects the change of agglomeration and heterogeneity of adjacent spatial units of local ESVs, and the Lisa diagram (Figure 9) shows that the spatial agglomeration of local units is influenced by the karst ecological restoration project. In order to coordinate the relationship between “economy and ecology” and promote the healthy development of the region, we should designate ecological red lines based on low–low, low–high and high–high aggregation areas, and implement parallel control and protection to control karstic rock desertification [46].

The results of the study showed that the conversion of grassland and cropland to woodland contributed the most to the increase in regional ecological asset value, with an overall average contribution of 68.46% for woodland, and that woodland expansion on grassland and cropland has become the dominant landscape type [47], which is similar to the findings of Yuan et al. [48]. Woodland expansion contributed 98.12% to the increase in ecological assets, which is much higher than the decrease in ecological assets caused by the conversion of woodland to cropland and cropland to construction land, so the overall ecological asset value is increased. However, in 2010–2018, the ecosystem service value of forest land appears to be flat, and the decrease in the area of cropland and grassland contributes the most to the decrease in ESVs, depriving the ecosystem service value of cropland and grassland conversion, and in order to reduce the loss of ecological assets, urbanization promotion should consider the optimization of ecological functions and coordinated economic development [49]. The landscape type shift from 1995–2018 did not change the contribution rate of single ecosystem service function value, soil formation and conservation, biodiversity conservation, gas regulation, water conservation and climate regulation remain important factors of ecological assets in karstic stone desert areas, but raw materials and recreation culture are more responsive to landscape type change and are the main factors of total service value fluctuation. Forests are the main landscape for raw materials, biodiversity, and carbon provision, but ecological conservation in karst areas should not focus only on forests, and grassland reduction has become the main aspect of net loss of carbon storage services [50].

This study suffers from the homogenization of remote sensing data, the degree of regional landscape fragmentation, and the blurred boundaries of each ecosystem type, resulting in the failure to clarify the transfer mechanism of secondary ecosystem service functions, negative service types, and system quality change patterns. The analysis of only natural landscape types may also cause a decrease in the accuracy of ecological asset value assessment. Therefore, we can strengthen the analysis of the impact of future intensification and structural transformation of landscape types on ecological assets, and need to establish a multi-factor-driven evaluation index system and methodological research on the spatial and temporal changes of ecological asset values, and identify the main control factors affecting services and functions and accurately assess the value of different landscape types. In this way we can lay the foundation for rational planning of ecological functional areas in the study area and assessing the effectiveness of ecosystem protection under rock desertification control.

## 5. Conclusions

Qixingguan District, Bijie City, Guizhou Province, is a typical representative of karst plateau mountains, and it is important to analyze the influence of its landscape type shift on the spatial and temporal evolution of ecological assets for the construction of karst ecological environments and regional economic development. The methodological analysis of sensitivity, spatial autocorrelation, and contribution rate showed that: (1) The three main landscape types of arable land, woodland, and grassland transform drastically and change the structure and function of the local ecosystem. (2) In time, the value of ecological assets showed a trend of increasing and then decreasing, and the expansion of woodland and the decline of grassland were the main reasons for their value fluctuations. (3) The overall spatial distribution of ecological assets has a significant positive correlation, and similar aggregation exists between adjacent units as a whole; local units have significant spatial differentiation characteristics. (4) Among the different landscape types shifted, the continuous expansion of woodland has the highest contribution to the value of ecological assets.

This paper carries out the spatial and temporal dynamic analysis of ecological asset value by using remote sensing follow technology, and clearly presents the spatial and temporal evolution mechanism of ecological assets based on the perspective of landscape type transfer. The interrelationship between different landscape types and different functions is analyzed through the dynamic change pattern of ecological asset value, exploring a new idea of clarifying the main control factors of regional ecological assessment and expanding the application framework of ecological asset accounting in ecological benefit assessment. It provides scientific basis for decision makers to reasonably plan landscape types and has important reference value for karst ecosystem restoration management, optimization of ecosystem service functions, and consolidation of stone desertification management effectiveness.

## Figures and Tables

**Table 1:** Ecosystem service value coefficients of landscape types in the study area (Yuan/hm^2^·a).

Ecosystem Service Functions	Arable Land	Woodland	Grassland	Waters	Construction Land	Unused Land
Gas regulation	705.71	4939.97	1129.14	0.00	0.00	0.00
Climate regulation	1256.16	3810.83	1270.28	649.25	0.00	0.00
Water conservation	846.85	4516.54	1129.14	28,764.74	0.00	42.34
Soil formation and protection	2060.67	5504.54	2752.27	14.11	0.00	28.23
Waste disposal	2314.73	1848.96	1848.96	25,659.62	0.00	14.11
Biodiversity conservation	1002.11	4601.23	1538.45	3514.44	0.00	479.88
Food production	1411.42	141.14	423.43	141.14	0.00	14.11
Raw material	141.14	3669.69	70.57	14.11	0.00	0.00
Entertainment culture	14.11	1806.62	56.46	6125.56	0.00	14.11

**Table 2:** Average annual change rates of landscape types in the study area during 1995–2018(%).

Landscape Type	1995–2000	2000–2005	2005–2010	2010–2015	2015–2018	Total Rate of Change
Arable Land	−0.11	0.42	−0.16	−0.27	−0.26	−0.06
Woodland	0.08	0.14	0.78	0.00	−0.05	0.21
Grassland	0.03	−1.04	−1.64	−0.12	−0.15	−0.60
Waters	−0.18	0.19	4.07	−0.11	0.14	0.88
Construction Land	5.55	−0.05	33.10	21.29	12.38	37.42
Unused Land	−2.25	0.04	−0.01	0.01	−0.12	−0.50

**Table 3:** Land use transition matrix in the study area from 1995–2018 (km^2^).

	2018						
1995	Arable Land	Woodland	Grassland	Waters	Construction Land	Unused Land	Amount of Change
Arable Land	1174.50	70.93	48.20	0.31	36.95	0.15	156.54
Woodland	75.24	1268.67	8.75	0.04	4.10	0.05	88.18
Grassland	60.99	83.61	556.27	0.09	10.46	0.13	155.28
Waters	0.07	0.02	0.06	1.22	0.01	0.00	0.16
Construction Land	0.36	0.02	0.05	0.00	5.50	0.00	0.44
Unused Land	0.83	0.09	0.10	0.00	0.00	4.98	1.01
Amount of change	137.49	154.67	57.16	0.43	51.52	0.33	401.60

**Table 4:** Global Moran’s I index of land use extent in the study area from 1995–2018.

Year	1995	2000	2005	2010	2015	2018
Moran’s I	0.4999	0.4970	0.4987	0.4892	0.4964	0.4963

**Table 5:** Changes in the value of individual ecosystem service functions in the study area from 1995–2018 (106 yuan).

	1995			2018			1995–2018	
Ecosystem Service Functions	ESVf	ELiT (%)	Rank	ESVf	ELiT (%)	Rank	∆ESVf	Rate of Change (%)
Gas regulation	844.59	13.58	3	864.98	13.71	3	20.38	2.41
Climate regulation	774.78	12.46	5	785.25	12.45	5	10.47	1.35
Water conservation	809.90	13.02	4	827.99	13.13	4	18.10	2.23
Soil formation and protection	1217.07	19.57	1	1222.69	19.39	1	5.62	0.46
Waste disposal	694.09	11.16	6	684.52	10.85	6	−9.57	−1.38
Biodiversity conservation	867.98	13.96	2	881.59	13.98	2	13.62	1.57
Food production	237.18	3.81	9	231.27	3.67	9	−5.91	−2.49
Raw material	521.76	8.39	7	545.17	8.64	7	23.41	4.49
Entertainment culture	251.89	4.05	8	263.48	4.18	8	11.59	4.60
Total	6219.23	100.00		6306.93	100.00		87.70	1.41

## Data Availability

The data presented in this study are available on request from the corresponding author.
